# RGD-Modified Nanofibers Enhance Outcomes in Rats after Sciatic Nerve Injury

**DOI:** 10.3390/jfb10020024

**Published:** 2019-05-29

**Authors:** McKay Cavanaugh, Elena Silantyeva, Galina Pylypiv Koh, Elham Malekzadeh, William D. Lanzinger, Rebecca Kuntz Willits, Matthew L. Becker

**Affiliations:** 1 Department of Biomedical Engineering, The University of Akron, Akron, OH 44325-0302, USA; mmc80@zips.uakron.edu (M.C.); gyp1@zips.uakron.edu (G.P.K.); em101@zips.uakron.edu (E.M.); 2 Department of Polymer Science, The University of Akron, Akron, OH 44325, USA; esilantyeva@zips.uakron.edu; 3 Cleveland Clinic Akron General, 1 Akron General Ave, Akron, OH 44307, USA; lanzinw@ccf.org

**Keywords:** peripheral nerve repair, RGD, aligned nanofibers, sciatic nerve, functionalized poly(*ɛ*-caprolactone), nerve guidance conduit, strain-promoted azide–alkyne

## Abstract

Nerve injuries requiring surgery are a significant problem without good clinical alternatives to the autograft. Tissue engineering strategies are critically needed to provide an alternative. In this study, we utilized aligned nanofibers that were click-modified with the bioactive peptide RGD for rat sciatic nerve repair. Empty conduits or conduits filled with either non-functionalized aligned nanofibers or RGD-functionalized aligned nanofibers were used to repair a 13 mm gap in the rat sciatic nerve of animals for six weeks. The aligned nanofibers encouraged cell infiltration and nerve repair as shown by histological analysis. RGD-functionalized nanofibers reduced muscle atrophy. During the six weeks of recovery, the animals were subjected to motor and sensory tests. Sensory recovery was improved in the RGD-functionalized nanofiber group by week 4, while other groups needed six weeks to show improvement after injury. Thus, the use of functionalized nanofibers provides cues that aid in in vivo nerve repair and should be considered as a future repair strategy.

## 1. Introduction

Peripheral nerve defects are serious and challenging health issues for surgical procedures in the fields of neurosurgery and plastic and orthopedic surgery [1], with an approximate incidence rate of 13 to 23 cases per 100,000 persons [2,3], primarily due to traumatic injury. Clinically, tension-free repair is paramount, and when anastomosis is not possible, nerve grafts or conduits must be utilized. In spite of being considered the gold standard of repair, autografts are often accompanied by shortcomings, such as sensory loss, scarring, denervation distal to the donor site, additional surgical procedures, and neuroma formation [4]. However, hollow nerve guidance conduits (NGC) are clinically approved alternatives to autograft repair, but their application is limited to a critical nerve gap of approximately 5 cm [4,5]. Cell-based tissue engineered alternatives have shown promising outcomes [6,7]; however, translational limitations for pathways with cells are long and complex. Acellular nerve grafts provide alternatives to nerve gap repair that involve extracellular matrix-like structures to encourage cell infiltration. However, acellular guidance conduits still demonstrate limited response in long gaps due to cell senescence [8]. Thus, our knowledge is still limited in the design of these intraluminal fillers to optimize neuroregeneration.

Cells play a critical role in the neuroregeneration process. For example, it has been demonstrated that Schwann cells (SC) play a critical role in guiding growing axons and providing the appropriate growth factor signals for regeneration [9], while macrophages and endothelial cells are important to the endogenous repair process by increasing SC infiltration [10]. Therefore, supporting SC migration into an acellular graft is an important step to consider in the development of intraluminal scaffolds [9,11,12]. A variety of cues have been used to enhance such a regenerative response, including electrical stimulation [13], mechanical stimulation [14], and topographical cues. The incorporation of intraluminal structures, such as aligned fibers, is a promising method to instigate the endogenous repair response, since fibers can serve as replacements for fibrin cables that are formed during the repair process and guide cell migration and axon outgrowth by providing topographical cues. [5] The alignment of nanofibers was found to be important, as aligned fibers improved regeneration over random nanofibers [15] in rat nerve defects. In addition, aligned nanoscale fibers support SC adhesion and proliferation *in vitro* [16] and SC migration in vivo [17]. Overall, previous work has clearly demonstrated an advantage of using aligned nanostructures to improve the endogenous repair process.

Along with topography-based cues, bioactive proteins or peptides can further improve nerve regeneration [18,19]. Laminin and its peptides, such as YIGSR and IKVAV, have been used as supportive additives to synthetic scaffolds for nerve regeneration [15,19,20,21]. The incorporation of these bioactive cues via chemical tethering allows for the control of concentration and spatial presentation compared to the use of physically adsorbed proteins. An efficient tool to chemically modify a polymeric fiber with peptides is strain-promoted azide–alkyne cycloaddition (SPAAC), a facile and quantitative post-fabrication modification chemistry requiring no catalyst or chemical activation. SPAAC has been used to fabricate YIGSR- [22,23] and RGD- [24] functionalized nanofiber substrates for neural differentiation *in vitro*. While RGD peptides have been shown to positively affect nerve regeneration [25,26], the method of integration of the peptide can alter the overall response, requiring further investigation of SPAAC integrated peptides.

In the current study, we sought to combine the use of bioactive RGD along with the topographical cue of nanofibers as guidance conduit intraluminal fillers in an in vivo rodent model of sciatic nerve defects. Over a six-week period, the motor and sensory functions of the rats were assessed. Histological analysis was performed to observe muscle atrophy and infiltration of SC and endothelial cells and other tissue regeneration processes. After six weeks, nanofiber groups showed SC infiltration and vascularization, with RGD functionalization significantly decreasing muscle atrophy and speeding sensory recovery. Thus, RGD functionalization of nanofibers facilitated regeneration in rat sciatic nerves over non-functionalized nanofibers.

## 2. Results

### 2.1. Nanofibrous Nerve Constructs

**Figure 1:**
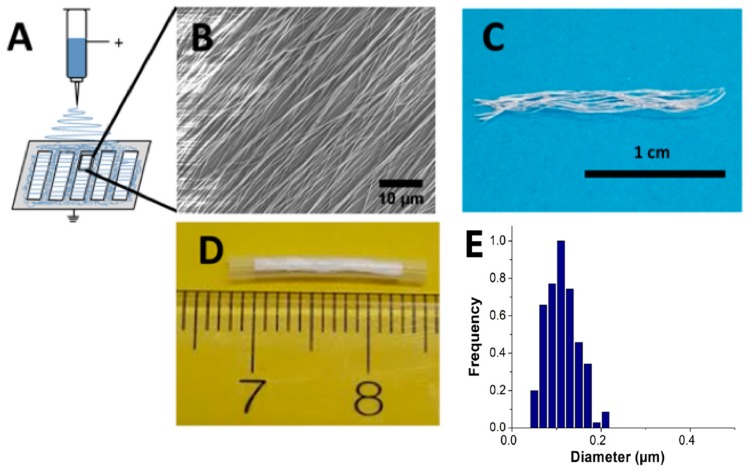
(**A**) Electrospinning setup for aligned nanofibers: a solution of 4-dibenzocyclooctynol (DIBO)-terminated poly(*ɛ*-caprolactone) in 1,1,1,3,3,3-hexafluoro-2-propanol (HFIP, 17% *w*/*v*) was placed in a syringe, and a voltage of 15 kV was applied to form aligned nanofibers in the gaps of a metal collector. (**B**) Analysis of SEM images was performed to estimate the topography of the nanofibers. (**C**) Aligned nanofibers were collected in the gaps of the collector by tweezers to form yarns. (**D**) Yarns were functionalized with RGD peptide, cut into 13 mm stripes, and placed inside a 17 mm silicone tube in a way that a 2 mm space was left on either side of the tube. For the control fiber group, the samples were placed in the tube without functionalization. (**E**) The distribution of fiber diameters (average diameter ᴓ = 112 ± 35 nm) was calculated using NIH ImageJ [27].

High-molecular-mass (*M*_n_ = 50.8 kDa, *M*_w_ = 68.6 kDa, *Đ*_M_ = 1.35) poly(*ɛ*-caprolactone) (PCL) functionalized with 4-dibenzocyclooctynol (DIBO) was synthesized via ring-opening polymerization of *ɛ*-caprolactone (yield = 77%; Figures S1 and S2a) and was used to fabricate aligned nanofibers. The average diameter of the fibers was determined to be ᴓ = 112 ± 35 nm (Figure 1) by SEM. Aligned nanofibers were rolled and functionalized, post-electrospinning, with GRGDS, referred to as RGD, as the active component, via strain-promoted azide–alkyne cycloaddition. The surface concentration of the peptide was determined to be ~16.1 pmol/cm^2^ by UV–VIS spectroscopy (Figure S2b).

### 2.2. Tissue Analysis

**Figure 2:**
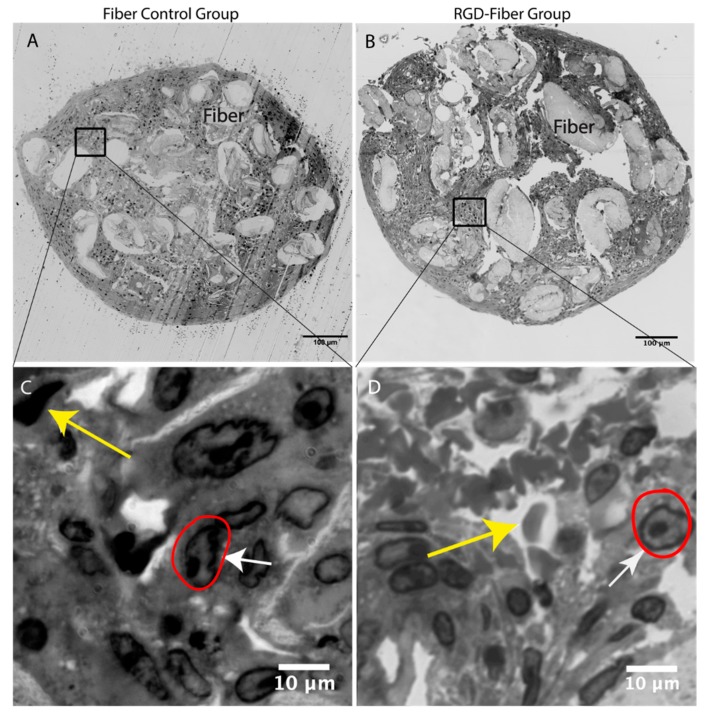
Toluidine blue staining of midline sections of (**A**) control fibers and (**B**) RGD-fibers imaged at 20× shows the presence of regenerated nerve tissue. Toluidine blue staining on midline sections imaged at 100× of (**C**) control fiber and (**D**) RGD-fiber samples (from boxed regions from A and B, respectively) showing myelinated axons (white arrow with red circle), fibers, and red blood cells (yellow arrow).

**Figure 3:**
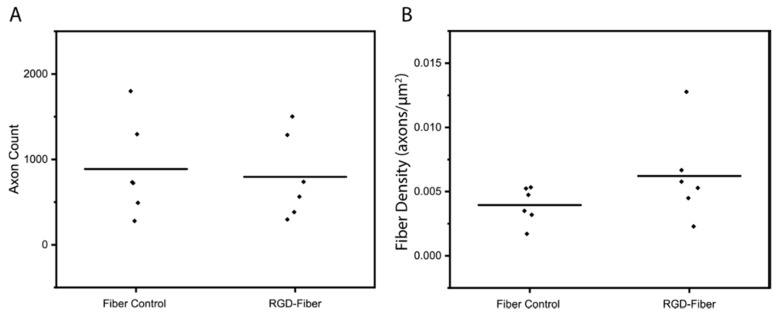
Axon count (**A**) from whole sections of toluidine blue-stained sections (n = 6 nerves). Control fibers were associated with an average of 886.5 ± 561.0 axons, and RGD-fiber with 794.7 ± 493.3 axons per whole nerve section. The groups were not significantly different. Axon fiber density (**B**) was 0.003953 ± 0.001412 axons/µm^2^ for the control fiber group, and 0.006214 ± 0.0003539 axons/µm^2^ for the RGD-fiber group. No significant differences were found between the groups. The total nerve sections used for the analysis can be seen in Figure S4.

From a gross evaluation of the injury site, no signs of neuroma formation or inflammation were observed in the area surrounding the repaired nerves. From a gross evaluation of the scaffold explants, no signs of degradation of the silicone conduit, control fibers, or RGD-fibers were detected at the time of sacrifice. Toluidine blue contrast staining revealed structural differences between the nerve sections from different groups. Sections from the midline (Figure 2) and at the interface of the implant with the tissue at the distal end (Figure S3) of both nanofiber groups were evaluated using 20× tile imaging on a light microscope to evaluate the overall structure. From the midline segments, a regenerative response was visible through axon growth within the nanofiber scaffolds (Figure 2A,B). The midline sections of RGD-fibers and control fibers showed some levels of tissue with visible myelinated axons. This tissue presence indicated that both of these groups had some proximal to distal connection at week 6. Midline sections were evaluated using 100× light microscopy to visualize structural details and to quantify the number of axons, which was used to calculate the fiber density. In both of the fiber groups, we saw evidence of red blood cells, indicating the presence of a blood supply at the midline (Figure 2C,D). No differences were found in axon count or fiber density between the fiber groups (Figure 3A,B).

**Figure 4:**
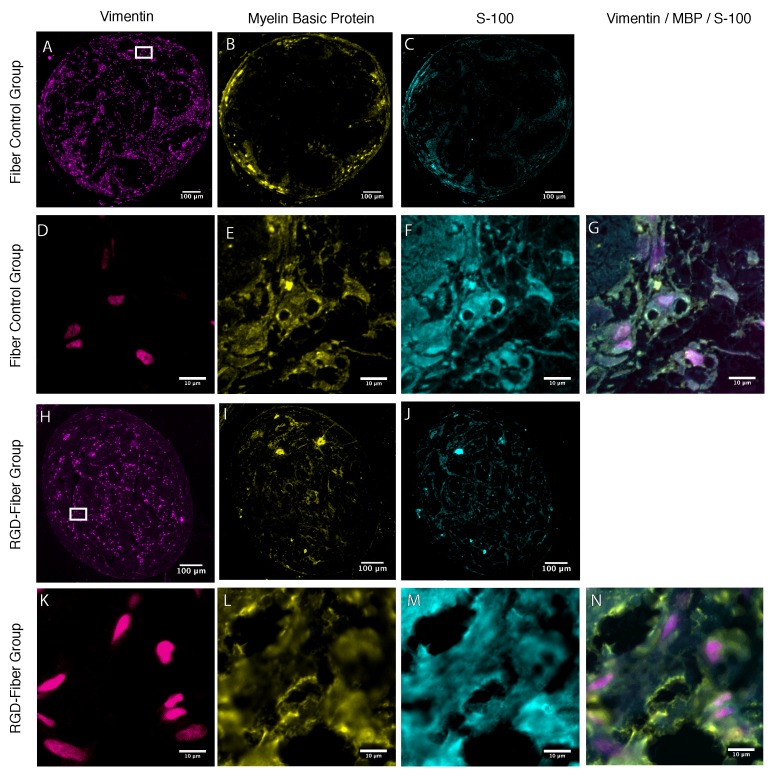
Immunohistochemical staining shows myelin basic protein (yellow), vimentin (magenta), and S-100 SC label (cyan) in midline sections from six-weeks samples of RGD-fibers (**H**–**N**) and control fibers (**A**–**G**). Both fiber groups showed similar presence of myelin basic protein (**B**,**E**,**I**,**L**), vimentin (**A**,**D**,**H**,**K**), and S-100 (**C**,**F**,**J**,**M**) at the midline, with myelin basic protein and S-100 labeling similar structures. Further magnified (100×) images from the boxed region in (**A**) and (**H**) for each group, respectively, to show specificity and overlap of the labels, including merged images at 100× (**G**,**N**).

Immunohistochemistry revealed no qualitative differences in cellular composition between the segments from each experimental group. Myelin basic protein (MBP), vimentin, and S-100 were expressed in the RGD-fiber samples and control fiber samples (Figure 4). Figure S5 displays controls for fluorescence with only secondary antibody at the same exposure times, thus showing the lack of nonspecific binding. To demonstrate specificity of S-100 and MBP staining, we overlaid the fluorescent images with a toluidine blue image of the same nerve; no labeling was found within blood vessels (Figure S5). On the basis of a visual evaluation, MBP had similar expression in RGD and control fiber samples, where it was expressed throughout the nerve segment and co-registered with S-100 (Figure 4G,N). In addition, the labeling of tissue with vimentin was more punctate and, at times, distinct from S-100 and myelin basic protein labeling. Analysis of the fluorescent images via ImageJ showed no significant differences between RGD-fibers and control fibers (Figure S6).

**Figure 5:**
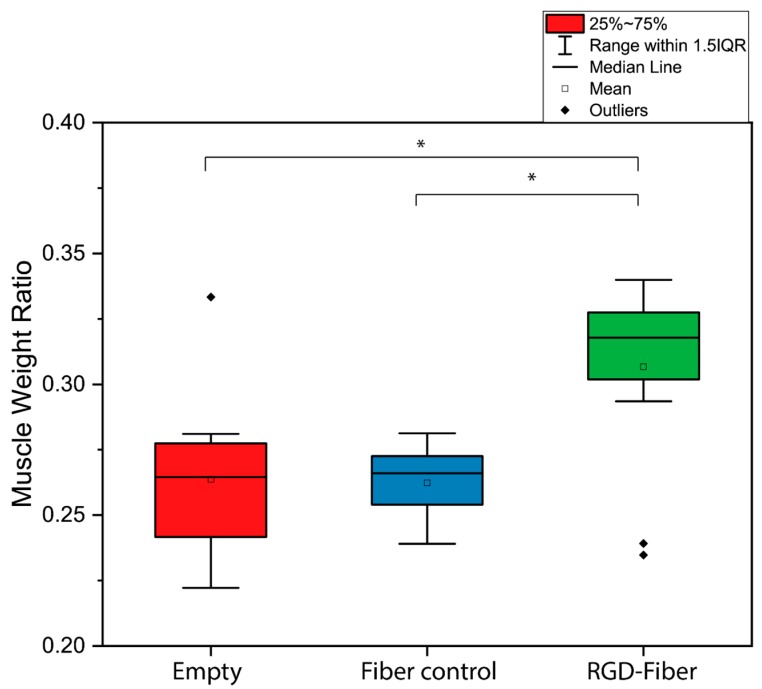
Muscle weight ratio at week 6. The RGD-fiber group had significantly less atrophy compared to the control fiber and to the empty conduit groups (*p* = 0.045 and *p* = 0.020). Significant differences were not found between the control fiber and the empty conduit groups. Outliers in the empty group (muscle weight ratio = 0.33) represented a conduit with nerve tissue formation via gross evaluation. (* denotes *p* < 0.05).

Muscle atrophy was determined by normalizing the weight of the experimental gastrocnemius muscle to that of the sham gastrocnemius muscle (Figure 5). RGD-fiber groups had less muscle atrophy at week 6 than control fiber groups or empty conduits (ratio of 0.307 ± 0.0348; 0.262 ± 0.0135; and 0.264 ± 0.0296, respectively), with statistical differences found between RGD-fiber and empty conduit (*p* = 0.020), and RGD-fiber and control fibers (*p* = 0.045). 

### 2.3. Functional Assessment

**Figure 6:**
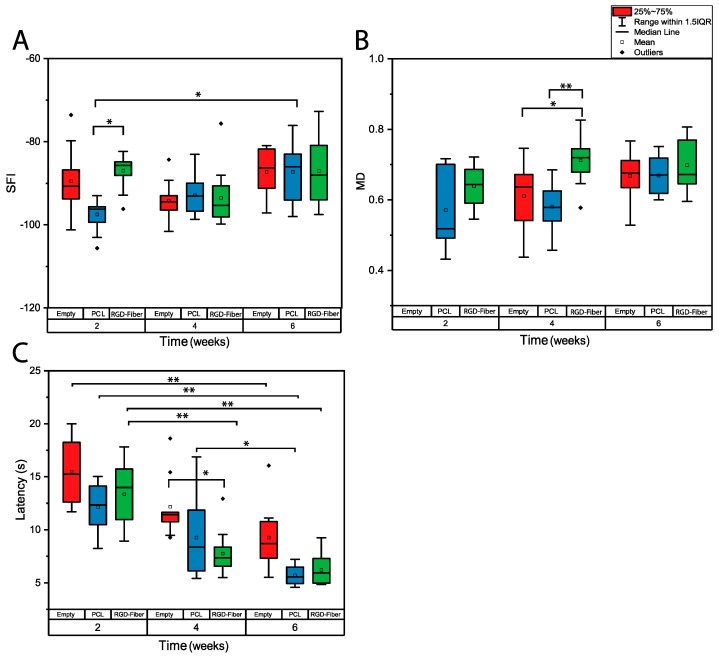
Motor and sensory functional data over the six weeks of the study. The sciatic function index (SFI) (**A**) and extensor postural thrust (EPT) (**B**) are presented for each group over time. As function improved, the SFI values became less negative. After two weeks, the RGD-fiber group was found to be significantly different from the control fiber group (*p* = 0.002). Animals with control fibers were found to improve significantly from week 2 to week 6 (*p* = 0.003). As function improved, the EPT values decreased. Significant differences were found between the RGD-fiber group and the empty conduit and control fiber groups at week 4 (*p* = 0.045 and *p* = 0.001, respectively). Sensory improvement (**C**) was demonstrated by a decrease in latency (s). As function improved, the latency decreased to pre-surgery values (Figure S7). In all groups, latency significantly decreased from two to six weeks (empty *p* ≤ 0.001, control fiber *p* ≤ 0.001, and RGD-fibers *p* ≤ 0.001); however, only the RGD-fiber group improved significantly from week 2 to week 4 (*p* ≤ 0.001). At week 4, animals with RGD-fibers showed significantly more recovery than those with empty conduits (*p* = 0.005). The control fiber group recovered significantly from week 4 to week 6 (*p* = 0.032) (* denotes *p* < 0.05 and ** denotes *p* ≤ 0.001).

The evaluation of sciatic function index (SFI) was performed every two weeks over six weeks post-surgery. After two weeks, animals with RGD-fibers had significantly less motor impairment (−87.0 ± 4.00) when compared to those with control fibers (*p* = 0.002; −97.5 ± 3.78). Rats with control fibers were found to significantly improve from week 2 (−97.5 ± 3.78) to week 6 (*p* = 0.003; −96.1 ± 7.26). At week 4, SFI was not found to differ between any groups (Figure 6A), although at week 6, the empty conduit group had a reduced number of animals (n = 5) due to a video failure.

Extensor postural thrust (EPT) provided another measure of motor performance after nerve injury and had lower standard deviation than SFI [28]. The forces generated by the experimental limb and the sham limb were recorded, and data are presented as the difference between the forces in the two legs normalized to that of the sham (muscle deficity ratio, MD). No statistical difference was found between the control fiber (n = 8) and RGD-fiber (n = 12) groups at week 2. Data were not collected at week 2 for the empty conduit group. By week 4, the RGD-fiber (0.712 ± 0.0653, n = 12) group had a significantly higher EPT compared to the empty conduit (n = 8) and control fiber groups (n = 12) (*p* = 0.045 and *p* = 0.001; 0.610 ± 0.106 and 0.581 ± 0.0667, respectively), but all groups were similar by week 6. Statistical differences were not found between any of the groups by week 6 (Figure 6B).

Sensory function was evaluated using a modified Hargraeves test, where response to a thermal stimulus via latency of paw retraction was measured in bi-weekly intervals during the six weeks of recovery. The baseline was set to ~4–7 s, and the animals demonstrated no variations in latency at time 0 (Figure S7). Sensory function was analyzed by the latency, measured in seconds, at each timepoint. All three groups improved significantly from week 2 to week 6, indicative of overall sensory recovery (empty conduit 15.5 ± 2.97 s to 9.25 ± 2.79 s, *p* ≤ 0.001; control fiber 12.1 ± 2.97 s to 5.70 ± 0.890 s, *p* ≤ 0.001; RGD-fibers 13.4 ± 2.78 s to 6.10 ± 1.40, *p* ≤ 0.001). However, the RGD-fiber group also improved significantly from week 2 to week 4 (13.4 ± 2.28 s to 7.74 ± 2.03 s, *p* ≤ 0.001) and at week 4, showed significantly more sensory improvement compared to the empty conduit group (7.74 ± 2.03 s, 12.2 ± 2.99 s; *p* = 0.005). The control fiber group was found to significantly improve from week 4 to week 6 as well (9.24 ± 3.63 s, 5.70 ± 0.890 s; *p* = 0.032) (Figure 6C).

## 3. Discussion

Several studies have shown the effect of natural and synthetic polymeric single hollow nerve conduits in axonal guidance and nerve regeneration to treat small gaps (<3 cm) [12]. The efficacy of these conduits decreased with the increase of nerve gap length [29]. Luminal fillers, including electrospun nanofibers, have been introduced as solutions to mimic fibrin cable formation, the aligned extracellular matrix bridge that serves as a topographical guidance for cell migration and nerve regeneration [5,30]. To further mimic the structure of native matrix that facilitates nerve regeneration, incorporation of bioactive molecules should be considered in the NGC design. In our study, the use of SPAAC resulted in efficient tethering of peptide to the surface of PCL nanofibers. While nanofibers functionalized with peptides via SPAAC have been previously used for directing stem cells [22,23,24] as well as Schwann cells [16] *in vitro*, the current study is the first effort to evaluate those substrates in vivo in a peripheral nerve injury model. With the ability to tether peptides onto nanofibers [16,22,24], we hypothesized that a longitudinally aligned scaffold functionalized with bioactive molecules would enhance nerve regeneration [31] and analyzed this assumption through functional tests and histological evaluations. As RGD had been previously shown to enhance SC adhesion and proliferation compared to other peptides [32], and SC are critical to nerve regeneration [9], we investigated if RGD functionalization would improve the neuroregenerative response compared to non-functionalized fibers.

A critical gap is a nerve defect that will not regenerate if unaided, estimated as >10 mm in the rat sciatic nerve injury model repaired with an empty silicone tube, due to the lack of the formation of a fibrin cable [18,33]. For this study, 3 out of 12 empty conduit samples, evaluated at week 6 after a 13 mm gap defect, had visible tissue by gross analysis at the midline, indicating some regeneration through the empty conduit. To determine if the tissue ingrowth was due to gap size error, the distance between sutures was measured on each empty conduit explant. When the suture distance measurements were found to be appropriate for all explants, the distance was not found to be correlated with the generation of nerve tissue (data not shown). This small rate of regeneration in the empty conduit group for a 13 mm gap injury was similar to that determined in previous works, [34,35] demonstrating the variability of the rodent model. Overall, this control was utilized to characterize the baseline response for rat nerve regeneration in the sciatic nerve model.

Histology was used to determine how the fibers supported a response at the tissue level. Toluidine blue-stained cross sections of the conduits revealed nerve tissue, with visible axons and vascularization at the midline. This tissue presence was indicative of nerve repair and blood supply by week 6, both pivotal parts of nerve regeneration and muscle reinnervation [36]. At the midline, quantitative evaluation of the histology sections showed that axon density was not different between the fiber groups at 6 weeks. As toluidine blue staining does not reveal cellular-level differences, immunohistochemistry was used to evaluate SC infiltration into the scaffolds. Our results demonstrated that SC were present at the midline of both fiber scaffolds after 6 weeks, which agreed with the presence of myelinated axons shown by toluidine blue staining. While others have demonstrated SC infiltration into grafts of self-assembled RGD peptide-adsorbed fibers after 12 weeks [26], our results showed evidence of infiltration after only 6 weeks (Figure 3). The difference might be related to fiber diameter, as larger microfibers were used previously (2 to 4.5 µm) [26], while we used nanofibers with smaller diameters (~112 nm). Myelin basic protein was also found in both nanofiber groups, indicating maturation of the SC through the early stages of myelination [37] (Figure 4). In native repair, SC along with fibroblasts, form the bands of Bünger, providing the framework for axonal regeneration. Vimentin was used as a label for fibroblasts [38] and found in all sections (Figure 4). However, vimentin is expressed on a variety of cells, including SC [39], so it was difficult to fully evaluate the presence or quantity of fibroblasts [40]. This variable expression can be seen in the overlap between vimentin labeling and S-100 labeling (Figure 4G,N); the presence of this overlap was shown in a mouse model to represent myelinating SC [39]. The literature also points to vimentin labeling during cell migration [41,42], which would be characteristic of both fibroblasts and SC at this stage of tissue reinnervation. The presence of these markers demonstrated that both nanofiber groups were providing guidance and support for SC infiltration and regenerative development within the conduit. Others have found differences in tissue marker expression between RGD-containing groups and controls after 12 weeks [26], thus, later than the 6 weeks required in this study. To better evaluate the differences between fiber groups, earlier time points may be necessary to provide a more thorough understanding of tissue development within the grafts and determine any differences throughout the time of repair.

While we did not find significant differences in nerve histology between the functionalized and non-functionalized fiber groups, after six weeks, muscle atrophy was found to be statistically lower in the RGD-fiber group than in the control group or the empty conduit control. However, this lower muscle atrophy in the RGD-functionalized group was not supported by the functional assessments. As motor recovery has proven difficult to measure in the past in short-duration studies, it was evaluated by two methods, SFI and EPT [28], which were used in combination with tissue analysis and other tests to show nerve recovery over time [28]. Specifically, SFI calculations have been shown to be highly variable at early recovery time points [43]. While the SFI test is less reliable at early time points [44], the SFI values found in the current study are consistent with those found in other nerve gap studies [21,25,26], and showed no differences between the groups at six weeks. While we did not find significant differences here for SFI, control fibers have been previously found to support less motor recovery, measured via SFI, compared to a bioactive peptide in conjunction with PCL over 12 weeks [26]. The EPT test provided another measure of motor function [28]. However, by week 6, no differences were found in EPT between the groups, and no differences between two- and six-week deficits. These results indicate that longer times may be necessary to see a functional improvement in motor tests, even when histological differences are present. Sensory recovery was seen in all groups over six weeks. However, the RGD-fiber group showed significantly faster recovery from week 2 to week 4 and also improved sensory response at week 4 compared to the empty conduit group. Therefore, the addition of bioactive groups to the fibers was found to speed sensory recovery, which agrees with previous studies showing that laminin used in conjunction with aligned nanofibers promoted better sensory function over 6 weeks in a rat tibial defect compared to unfunctionalized fibers, although aligned nanofibers alone also induced significant improvements in sensory function [15]. Overall, nanofibers support functional recovery in sensory evaluations, but the addition of RGD seems to speed the response. Future work should focus on the identification of the specific regenerating axons to specifically match the functional response to the histological evaluation.

In summary, RGD-tethered nanofibers showed improved sensory function after four weeks as well as reduced muscle atrophy. After six weeks, both fiber groups had SCs present at the midline as well as organized myelin basic protein in addition to vimentin labeling. Overall, these results indicated that the addition of RGD to aligned PCL nanofibers improved nerve regeneration both functionally and histologically by improving initial motor recovery and decreasing muscle atrophy. However, it is clear that the topography was important in the process, as both groups showed nerve regeneration and cell infiltration.

## 4. Materials and Methods

### 4.1. Materials

All materials were used as received unless otherwise stated. Chloroform (anhydrous, containing amylenes as a stabilizer, ≥99%) and calcium hydride (reagent-grade, 95%) were purchased from Sigma-Aldrich (St. Louis, MO, USA). Methylene chloride was purchased from Fisher Scientific (Houston, TX, USA); 1,1,1,3,3,3-hexafluoro-2-propanol (HFIP) was purchased from Oakwood Products, Inc. (Estill, SC, USA). Methanol (ACS grade) and hydrochloric acid (36.5–38%, ACS Grade) were purchased from VWR International (Radnor, PA, USA). Dry toluene (HPLC Grade, 99.7%, Alfa Aesar) for polymerization was purified and dried on an Inert Pure Solv system (MD Solvent Purification system, model PS-MD-3, Amesbury, MA, USA) and degassed using three cycles of freeze–vacuum–thaw. *ε*-Caprolactone (*ε*-CL, 99%, ACROS Organics™, Thermo Fisher Scientific, Waltham, MA, USA) was dried over calcium hydride under nitrogen overnight and distilled under reduced pressure. Magnesium and 4-dibenzocyclooctynol (DIBO) initiator 2,6-di-tert-butyl-4-methylphenoxide catalyst [Mg(BHT)_2_(THF)_2_] were synthesized using methods described previously [45,46,47,48]. N_3_-GRGDS peptide was purchased from AnaSpec Inc., Fremont, CA, USA. Hollow silicone tubing (inner diameter = 1.7 mm, outer diameter = 2.0 mm) was purchased from Specialty Manufacturing, Inc., Saginaw, MI, USA.

### 4.2. Nerve Guidance Conduits Fabrication Methods

Proton ^1^H NMR (300 MHz and 500 MHz) spectra were recorded on Varian Mercury 300 and 500 spectrometers. The polymer was dissolved in CDCl_3_ solvent at 15 mg/mL, the relaxation time was 2 sec with 64 transients. Size exclusion chromatography (SEC) was used to determine molecular mass and molecular mass distributions (*Đ*_M_). Chromatograms were collected on a Tosoh EcoSEC HLC-8320GPC with refractive index detector, using *N,N*-dimethylformamide (DMF) containing 0.1 M lithium bromide as the eluent. The two columns were calibrated using narrow-molecular-mass polystyrene standards (20 standards from 0.5 Da to 5480 kDa), the flow rate was 0.3 mL/min at 40 °C. A high-voltage power supply (ES30P-5W, Gamma High Voltage, Ormond Beach, FL, USA) was used for electrospinning. The nanofiber scaffolds were characterized using a scanning electron microscope (SEM) with an applied voltage of 5 kV (JSM-7401F, JEOL, Peabody, MA, USA).

**Scheme 1:**
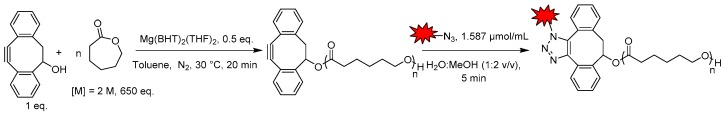
DIBO-end-functionalized poly(*ɛ*-caprolactone) was synthesized by ring-opening polymerization of *ɛ*-caprolactone with DIBO as initiator and was modified post-electrospinning with RGD peptide via strain-promoted azide–alkyne cycloaddition (SPAAC).

DIBO-end-functionalized poly(*ɛ*-caprolactone) was synthesized (Scheme 1), electrospun, and modified with GRGDS peptide using methods described previously [24]. Briefly, using standard drying techniques, an ampoule was filled with *ɛ*-CL (0.3340 mol, 37.01 mL), DIBO (0.5148 mmol, 113.25 mg), toluene (120.93 mL), and Mg(BHT)_2_(THF)_2_ (0.285 mmol, 173.00 mg). The ampoule was sealed and heated at 30 °C for 20 min. The polymerization was quenched with the addition of acidified (5% *v*/*v* HCl) methanol, dissolved into chloroform, and precipitated into cold methanol. The crude polymer was re-dissolved in methylene chloride and dried under high vacuum. The purified polymer was then stored in a desiccator. The monomer conversion (~100%) and the product (yield 77%) were confirmed by ^1^H NMR spectroscopy (Figure S1), UV–visible spectroscopy, and SEC (Figure S2, *M*_n_ = 50,800 Da, *M*_w_ = 68,600 Da, *Đ*_M_ = 1.35).

Aligned nanofibers were fabricated via electrospinning of the DIBO-terminated PCL solution in HFIP (17% *w*/*v*). The solution was placed in a 2 mL glass syringe with a 22-gauge needle (Jensen Global Dispensing Solutions, Santa Barbara, CA, USA), and a voltage of 15 kV was applied. Aligned nanofibers were collected in the gaps of the collector, placed at 10 cm from the tip by tweezers to form yarns (Figure 1C). Yarns were modified with RGD peptide via strain-promoted azide–alkyne cycloaddition by dipping into a solution of the N_3_-GRGDS peptide (1.587 µmol/mL) in a 1:2 water/methanol (*v*/*v*) solution for 5 min, rinsed with a 1:2 water/methanol (*v*/*v*) solution, blown with nitrogen, and dried overnight in a desiccator. Yarns were cut into 13 mm strips and placed inside of a 17 mm hollow silicone tube in a way that a 2 mm space was left on each side of the tube (Figure 1D). For the control fiber group, the samples were placed into the tube without modification.

Samples for SEM analysis were collected on glass slides, fixed with a double-side conductive tape to a metallic stud, and sputter-coated for 30 s with silver under a nitrogen atmosphere prior to imaging. The variation in nanofiber diameters was measured on at least three independent samples (five images of each sample with ≥50 fibers per sample) using NIH ImageJ [49] and reported as an average ± standard deviation. The extent of functionalization with peptide was confirmed using UV−visible spectroscopy with spectral resolution 1 nm using chloroform as a solvent. The peak intensity at 306 nm (which corresponds to the π-π* transition in the alkyne bond in DIBO-functionalized polymer) decreased after reaction with the azide-functionalized peptide in comparison with fibers before functionalization.

Nerve guidance conduits were sterilized by ethylene oxide using an Anprolene benchtop sterilizer (Anderson Products, Inc., Haw River, NC, USA) according to the manufacturer’s protocol for 12 h at room temperature and 35% humidity (the concentration of ethylene oxide was about 0.5 g/L), purged for 48 h, and stored in a vacuum desiccator until used for surgeries.

### 4.3. Experimental Design and Animals

All procedures and experiments involving animals were reviewed and approved by the Institutional Animal Care and Use Committee at The University of Akron. A total of 36 male Lewis rats from Envigo, weighing on average 262 ± 35.17 g each, were utilized in the study. The rats were randomly divided into three experimental groups: empty conduit (n = 12) as the negative control (empty silicone tube); control fibers (n = 12) as non-functionalized aligned-nanofiber grafts; and RGD-fibers (n = 12) as RGD-functionalized aligned-nanofiber grafts. The animals were housed in pairs in cages with an ambient temperature of 69–79 °F, 30–70% air humidity, and a 12 h day/night cycle. The rats had free access to standard rodent laboratory food (at least 5 g food per 100 g of animal’s body weight per day) and water. The animals were analyzed for functional recovery metrics involving both sensory and motor tests every two weeks after surgery over the course of the six-week study.

#### 4.3.1. Surgical Procedures

All animals underwent a surgical procedure to expose the sciatic nerves of both legs. A cutaneous incision was made directly below the acetabulofemoral joint, and an intermuscular plane parallel to the femur was separated to expose the sciatic nerve. For each animal, a randomly chosen leg served as a sham, where the sciatic nerve was exposed but remained uninjured. In the contralateral leg, which served as the experimental leg, a nerve segment was excised. A 2 mm length of each nerve stump was positioned inside the ends of the 17 mm silicone tube NGC and sutured in place, creating a 13 mm end-to-end gap defect. The muscle layers, which were separated during dissection, were gently pulled back together, and sterile Michel clips were used to close the skin. Recovery was carried out on a heated pad for observation of breathing and mobility until full consciousness and motion were restored, whereupon the animal was returned to its cage. The animals were inspected on a daily basis for mobility and evidence of pain or discomfort. Wound status was examined until the Michel clips were removed.

#### 4.3.2. Tissue Analysis

Gastrocnemius muscles were harvested after six weeks and were utilized to assess muscle maturity over the time period of nerve recovery. Muscles were harvested from both legs, fixed in 4% paraformaldehyde for 48 h at 4 °C, and then stored in sterile PBS with 1% penicillin streptomycin and 0.01% sodium azide at 4 °C. Wet muscle weights were recorded using an electronic balance, and the muscle weight ratio was calculated using the experimental side weight and the sham side weight.

Sciatic nerve explants were utilized to evaluate histological differences. After six weeks, six random nerve samples from each group were processed for histological analysis. The samples were post-fixed in 2% buffered paraformaldehyde and 2% glutaraldehyde. After fixation, the samples were embedded in polybed epoxy, sectioned into 500 nm sections on an ultramicrotome, and placed on superfrost charged slides. The samples with a conduit were cut at the estimated midline and at the nerve–scaffold junction of the distal end. The remaining six samples from each group were kept in 2% buffered paraformaldehyde and 2% glutaraldehyde and saved for additional analysis of unembedded tissue, which proved to be unsuccessful.

Multiple slides from each sample were prepared. Contrast staining was performed by applying toluidine blue stain to midline and distal sections. Whole nerve sections were imaged at 20× to create tile images to observe the overall structure of the samples at the midline and distal ends (Figure S4). Samples of midline sections were imaged at 100× to observe structural details (n = 6 nerves). For each nerve, a 100× tile image of the entire section was used for axon counting using ImageJ analysis tools [27]. Axon fiber density was calculated by taking the total axon count from the midline sections and dividing by the tissue area. Tissue area was determined by outlining the area of the entire section and subtracting the area containing fibers [27,35,49].

To prepare the slides for immunohistochemistry, sections were processed for antigen retrieval, plastic removal and rehydrated following a procedure adapted from the literature [50]. The slides were soaked in sodium ethoxide for 15 min to remove the surrounding polybed solution from around the nerve tissue. Next, the samples were rehydrated by soaking in a series of ethanol dilutions with decreasing concentrations, including 100%, 95%, 70%, 50%, and 25% ethanol. Antigen retrieval was performed by boiling the slides in a 0.01M (pH = 6) citrate buffer for a total of 20 min in a 700 W microwave, at 5 min intervals [50]. After boiling, the slides were left to cool for 30 min, while submerged in the citrate buffer.

Immunohistochemistry was performed to identify the expression of myelin basic protein (MBP), vimentin, and S-100. After antigen retrieval, the samples were blocked with 7.5% bovine serum albumin for 30 min and rinsed with 1× PBS for 5 min, and primary antibodies were applied at 4 °C overnight. Each experimental group had a minimum of three control sections, which were not exposed to primary antibodies but were exposed to secondary antibody. After primary antibodies, the samples were washed with 1× PBS three times for 5 min each before applying secondary antibodies. The antibodies for S-100 intracellular protein, a common label for SC, were anti-S-100 rabbit polyclonal IgG primary antibody (MilliporeSigma, SC644, St. Louis, MO, USA) and donkey anti-rabbit IgG Alexa Fluor 647 secondary antibody preadsorbed for mouse (Abcam, ab150063, Cambridge, MA, USA). The antibodies for myelin basic protein were mouse anti-myelin basic protein monoclonal IgG2b (Abcam, ab62631, Cambridge, MA, USA) and goat anti-mouse IgG2b Alexa Fluor 488 secondary antibody preadsorbed against mouse IgG1 (ThermoFisher, A-21141, Rockford, IL, USA). The antibodies for vimentin were mouse anti-vimentin monoclonal IgG1 primary antibody (Abcam, ab8978, Cambridge, MA, USA) and goat anti-mouse IgG1 Alexa Fluor 546 secondary antibody preadsorbed against mouse IgG2b (ThermoFisher, A-21123, Rockford, IL, USA). Finally, the sections were washed four times with 1× PBS for 5 min per wash, rinsed with DI water, and dehydrated by applying a series of ethanol dilutions with increasing concentrations, including 25%, 50%, 70%, 90%, and 100% ethanol. The slides were dried fully and mounted using Fluoromount^®^ (Thermo Fisher Scientific, Waltham, MA, USA) and coverslips. The mount set overnight and was sealed around the edges the next day to prepare for imaging. Each section had a control, treated only with secondary antibody and used during the analysis to determine background florescence. Entire sections were imaged at 20× using fluorescent microscopy to observe protein expression. All images were taken at the same magnification using the same exposure times, and a control with only secondary antibody was utilized to set a maximum exposure per slide (see Figure S4). The images were analyzed by randomly selecting three images per section, removing the background fluorescence determined by the control well, and then determining the average intensity per area section using ImageJ (n = 3) [49].

#### 4.3.3. Functional Assessment

Functional recovery tests were performed biweekly over the sis weeks of recovery from surgery on each rat. 

##### Motor Recovery

Motor recovery was assessed using two complementary methods, sciatic functional index (SFI) and extensor postural thrust (EPT) [28]. For SFI, paw prints were recorded. The following measurements were collected: print length (distance from the heel to the third toe; PL), toe spread (distance from the first to the fifth toe; TS), and intermediate toe spread (distance from the second to the fourth toe; IT). These values were taken from both the uninjured normal limb (NPL, NTS, and NIT) and the surgically injured experimental limb (EPL, ETS, and EIT). These measurements were used to calculate SFI using Equation (1), based on the equation developed by De Medinaceli et al. [51] and adapted by Bain et al. [52].

SFI = (−38.3 × PLF) + (109.5 × TSF) + (13.3 × ITF) − 8.8
(1)

where PLF = (EPL − NPL)/NPL; TSF = (ETS − NTS)/NTS, and ITF = (EIT − NIT)/NIT.

SFI score ranged from −8.8 to −100, with −8.8 indicating no differences between injured and non-injured paw, and −100 indicating total impairment.

Motor performance was also examined using EPT testing. The entire body of the rat, except one hind-limb, was gently wrapped in a surgical towel with the free hind-limb extending out. The animal was placed over the platform of a digital balance. Once the animal made a contact with the scale between the distal metatarsus and digits, the force in grams was recorded. The force generated by the animal was measured five times per rat, and the largest three observations were used for motor performance evaluation; this was done for each rat at time points of four and six weeks. The difference in force was normalized for each rat at each timepoint using the Equation (2), where NEPT is the normal uninjured limb, and EEPT is the experimental injured limb, as described by Koka and Hadlock [28]. The MD decreases to 0 as motor performance improves.

MD = (NEPT − EEPT)/NEPT
(2)

##### Sensory Recovery

The sensory tests of the plantar surface of the paw were conducted with a device developed on the basis of the Hargreaves method using unilateral hind paw withdrawal latency to radiant heat stimulation [53]. As the animals were unrestrained, they were given time to acclimate to their enclosure prior to testing. The rats were placed in clear plexiglass rectangular chambers (12 × 16 × 30 cm^3^) on a glass shelf (2-mm thick). An indicator light was used to locate the center of the rat’s hind paw. A switch simultaneously activated both the radiant heat and light sources, as well as an electronic timer, which was used to determine the exposure time until the paw was retracted; this was the latency value. A thermal lamp was held beneath the plantar surface of the paw to be tested until retraction of the paw, after which the latency value was recorded. If the rat did not respond to the heat stimulus, the thermal lamp would automatically shut off at the cutoff time of 20 s. For all animals, the time delay between heat exposure of the right and left paws was randomly selected to avoid errors caused by subject adaptation. The latency was measured in triplicate for all groups at all time points throughout the six-week testing period. Latency is reported in seconds for the experimental limb at each timepoint.

#### 4.3.4. Statistical Analysis

Experimental results were expressed as mean ± standard deviation, and *p* < 0.05 was considered significant. Differences between groups for SFI over the six-week study were evaluated using a general linear model (GLM), and multiple/post hoc group comparisons were performed by Bonferroni test. The GLM procedure is an ANOVA using a least-squares regression approach to perform multiple comparisons. Differences between groups for EPT for week 2 were evaluated using one-way ANOVA, due to the lack of empty conduit data; for the rest of the timepoints, differences between time and groups were evaluated with GLM, and group comparisons were made using Bonferroni test. Differences between groups over the six-week study for sensory testing were evaluated using GLM, and comparisons between groups were done by Bonferroni test. The relative gastrocnemius muscle weight measurements were performed using a non-parametric Kruskal–Wallis test, as the data were not normally distributed, and multiple/post hoc group comparisons were done by Dunn’s test with Bonferroni adjustment [54]; outliers were removed prior to statistical analysis. Differences between groups for fluorescent immunohistochemistry labeling, axon count, and axon fiber density were determined using the Student’s t-test.

## Supplementary Materials

The following are available online at https://www.mdpi.com/2079-4983/10/2/24/s1, Figure S1: ^1^H NMR spectrum of DIBO-terminated poly(*ɛ*-caprolactone), Figure S2: (A) DMF size-exclusion chromatography of DIBO-terminated poly(*ɛ*-caprolactone), (B) UV–visible spectrometry was used to measure the concentration of GRGDS peptide by comparison of absorbance at 306 nm corresponding to π-π* transition of the strained alkyne in DIBO. Figure S3: Toluidine blue stained images of distal nerve sections, (a) control fibers and (b) RGD-fibers imaged at 20×, showing the presence of myelinated axons, red blood cells, and fibers. Figure S4: Toluidine blue-stained images of midline nerve sections used for total axon count and density, (a) control fibers and (b) RGD-fibers imaged at 20×. Figure S5: Because of antigen retrieval, sections were exposed to harsh environments. Therefore, controls were performed for both nonspecific binding (left) and specificity of the primary antibodies (right). (LEFT) Control images used for fluorescent quantification to show the lack of non-specific binding of the secondary antibodies used. (RIGHT) Validation of S-100 and MBP labeling shown by matching a plastic embedded section for toluidine blue staining with an antigen-retrieved section labeled for S-100 and MBP. The lack of labeling within the blood vessels (green arrow heads) indicated that both S-100 and MBP were specific for their respective proteins. Figure S6: Fluorescent analysis of 20× images of midline sections showed the presence of MBP, vimentin, and S-100. Statistical differences were not found between control (n = 4) and RGD- (n = 3) fiber samples. Figure S7: Pre-surgery latency, measured in seconds, for the experimental leg, demonstrating that all animals had similar latencies at the start of the experiments.
