# Supplementary material for: RGD-Modified Nanofibers Enhance Outcomes in Rats after Sciatic Nerve Injury
Source: J Funct Biomater. 2019 May 29;10(2):24. doi: 10.3390/jfb10020024 (PMC6637389; doi:10.3390/jfb10020024)
Supplement: Supplementary file 1 [file jfb-10-00024-s001.pdf]

Journal of *Functional Biomaterials*

Article

RGD-Modified Nanofibers Enhance Outcomes in Rats after Sciatic Nerve Injury

McKay Cavanaugh [1], Elena Silantyeva [2], Galina Pylypiv Koh [1], Elham Malekzadeh [1], William D. Lanzinger [3], Rebecca Kuntz Willits [1,*] and Matthew L. Becker [2,*]

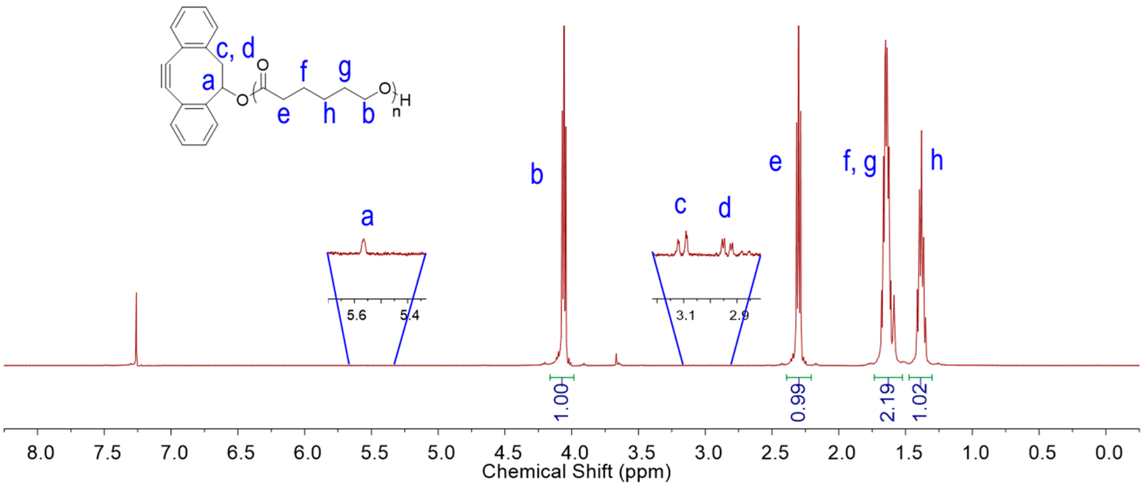

Figure S1. ^{1}H NMR spectrum of DIBO-terminated poly(ε-caprolactone) (500 MHz, $CDCl_3$, 303 K) confirms successful synthesis of polymer and survival of DIBO groups after polymerization. ^{1}H NMR (500 MHz, $CDCl_3$, 303 K): δ = 7.51–7.45 (m, aromatic), 5.56 (dd, $^3J_{H\text{-}H}$ = 3.2, 2.5 C*H*OH), 4.15–3.98 (m, $CH_2CH_2OCH_2$), 3.10 (dd, $^3J_{H\text{-}H}$ = 15.2, 2.1 Hz, C*H*(H)CH), 2.93 (dd, $^3J_{H\text{-}H}$ = 15.1, 3.9 Hz, CH(*H*)CH), 2.38–2.22 (m, (C=O)CH_2CH_2), 1.72–1.54 (m, (C=O)$CH_2CH_2CH_2CH_2CH_2$), 1.45–1.28 (m, (C=O)$(CH_2)_2CH_2((CH_2)_2)$ ppm.

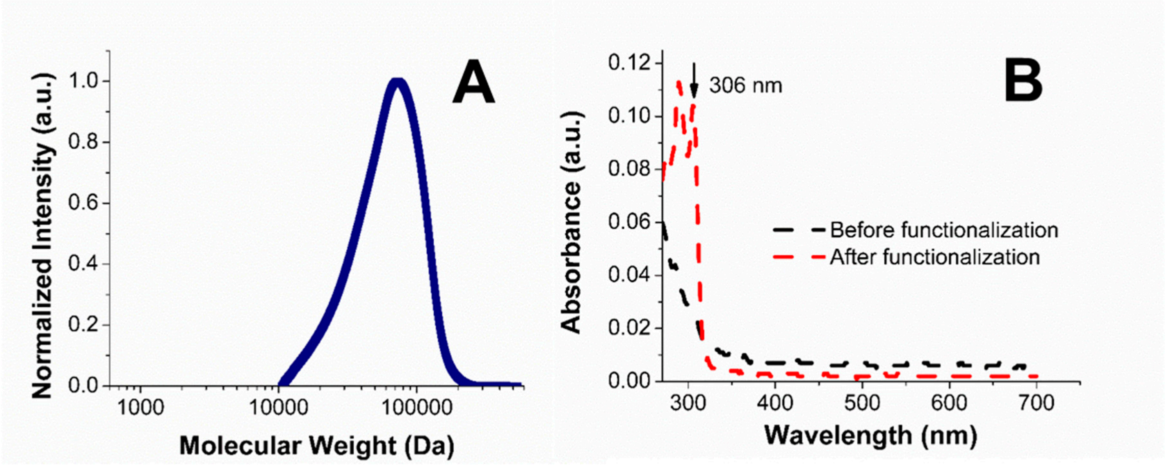

Figure S2. (**A**) DMF size exclusion chromatography confirms successful synthesis of high molecular mass DIBO-terminated poly(ε-caprolactone) (M_n = 50.8 kDa, M_w = 68.6 kDa, $Đ_M$ = 1.35). (**B**) UV-visible spectroscopy was used to measure concentration of GRGDS peptide by comparison of absorbance at 306 nm corresponds to π–π* transition of the strained alkyne in DIBO.

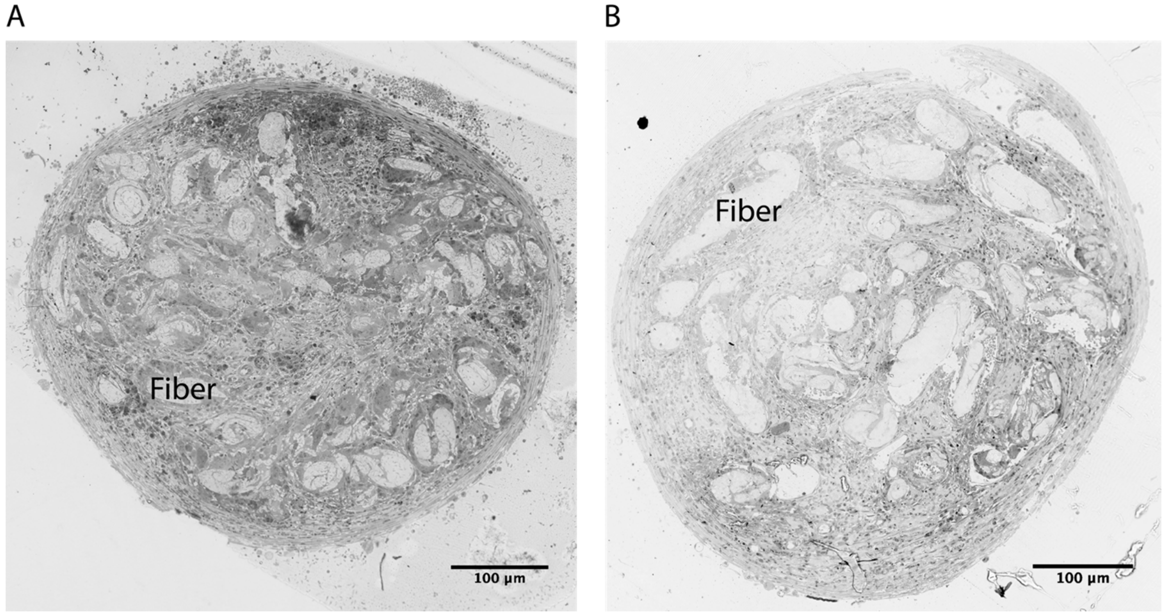

Figure S3. Toluidine blue stained images of distal nerve sections (**A**) control fibers and (**B**) RGD-fibers imaged at 20×, showing the presence of myelinated axons, red blood cells, and fibers.

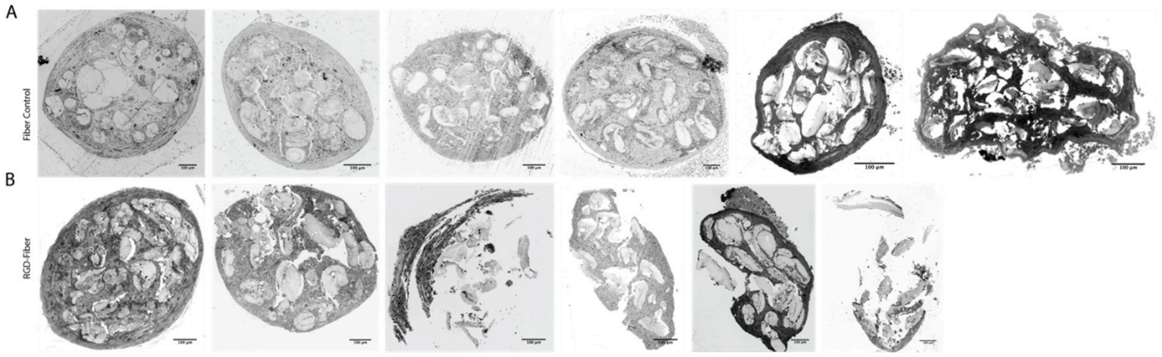

Figure S4. Toluidine blue stained images of midline nerve sections used for total axon count and density (**A**) control fibers and (**B**) RGD-fibers imaged at 20×.

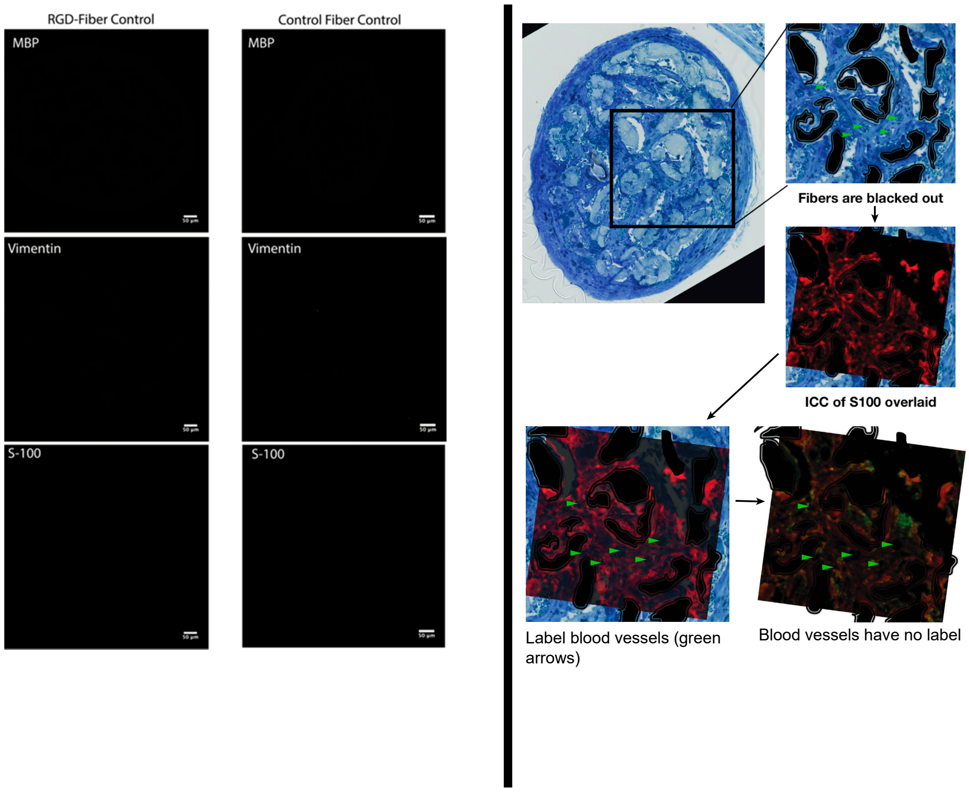

Figure S5. Due to antigen retrieval, sections were exposed to harsh environments. Therefore, controls were performed for both nonspecific binding (left) and specificity of the primary antibodies (right). (LEFT) Control images used for fluorescent quantification to show the lack of non-specific binding of the secondary antibodies used. (RIGHT) Validation of S-100 and MBP labeling shown by matching a plastic embedded section for toluidine blue staining with an antigen retrieved section labeled for S-100 and MBP. The lack of labeling within the blood vessels (green arrow heads) indicated that both S-100 and MBP were specific for their respective proteins.

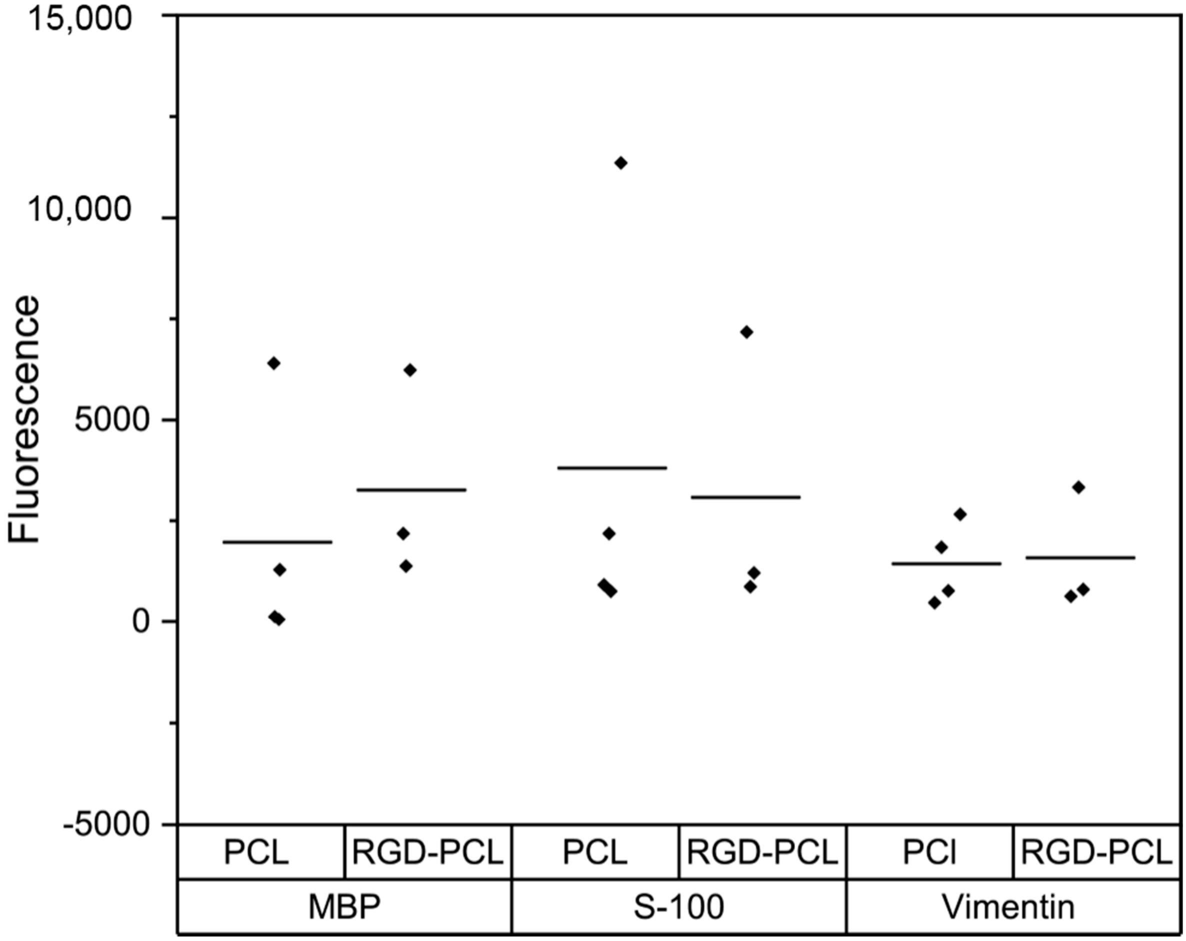

Figure S6. Fluorescent analysis of 20× images of midline sections showed the presence of MBP, vimentin and S-100. Statistical differences were not found between control (n = 4) and RGD- (n = 3) fiber samples.

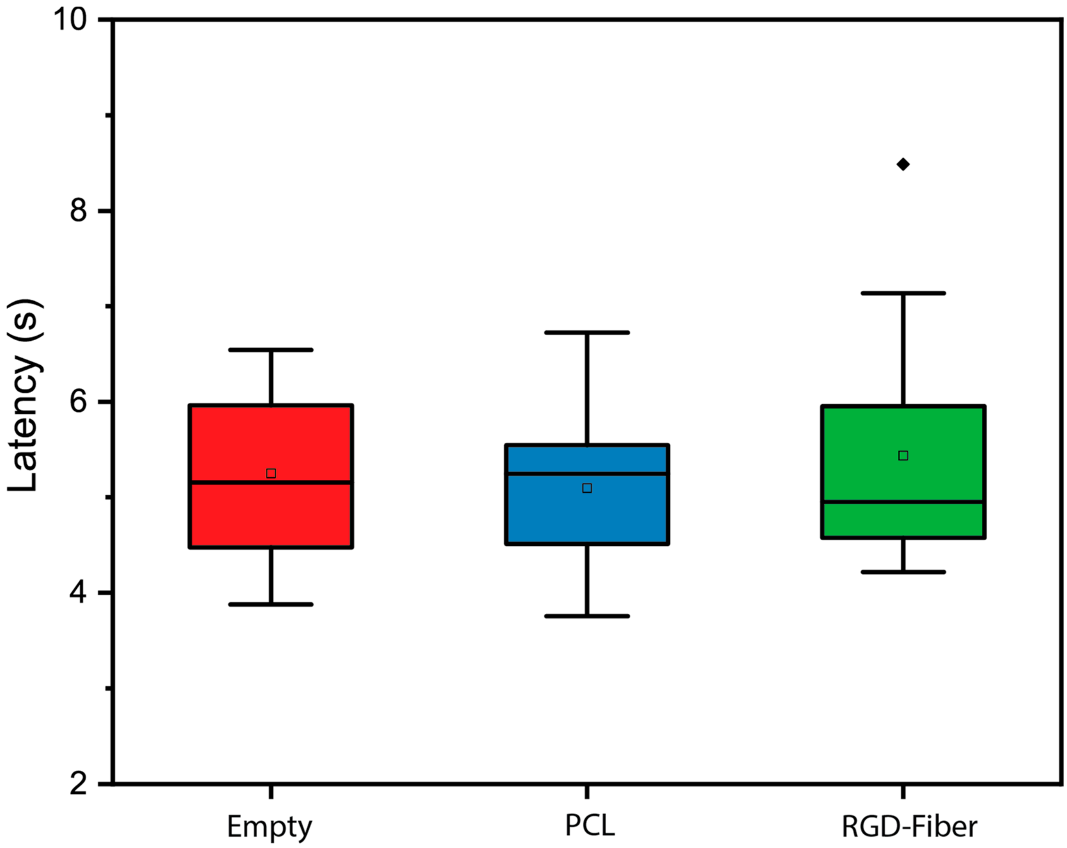

Figure S7. Pre-surgery latency, measured in seconds, for the experimental leg, demonstrating that the animals all had similar latencies at the start of the experiments.
